# Papillary cystadenocarcinoma of the parotid gland: a rare sub-variant of salivary gland adenocarcinoma

**DOI:** 10.4322/acr.2021.357

**Published:** 2022-02-24

**Authors:** Satya Dutta, Biswajit Dey, Jaya Mishra, Vandana Raphael, Jonali Das, Donboklang Lynser

**Affiliations:** 1 North Eastern Indira Gandhi Regional Institute of Health and Medical Sciences, Department of Pathology, Mawdiangdiang, Shillong, India; 2 North Eastern Indira Gandhi Regional Institute of Health and Medical Sciences, Department of Radiology, Mawdiangdiang, Shillong, India

**Keywords:** Papillary cystadenocarcinoma, Salivary gland neoplasm, Parotid

## Abstract

Papillary cystadenocarcinoma of the salivary gland is a very rare malignant neoplasm accounting for only 2% of all salivary gland lesions. In 1991 it was first included as a separate entity in the World Health Organization (WHO) classification of salivary gland tumors and in 2017 WHO Classification, the tumor was clubbed as a sub-variant of adenocarcinoma, not otherwise specified. It most commonly occurs in the major salivary glands. Herein we report a case of salivary papillary cystadenocarcinoma in a 54-year-old female, who presented with rapid enlargement of the right parotid swelling. Based on radiology and fine-needle aspiration cytology, a working diagnosis of the malignant tumor involving the superficial lobe of the right parotid gland was made. In view of the malignant nature of the swelling, superficial parotidectomy was done. The histopathology and immunohistochemistry of the mass confirmed the diagnosis of papillary cystadenocarcinoma of the right parotid. With the revised 2017 WHO classification of salivary gland tumors, it is important to report all rare subtypes in order to understand their biology and behavior.

## INTRODUCTION

In 1991, the papillary cystadenocarcinoma was considered as a separate entity in the World Health Organization (WHO) classification of tumors of the salivary glands.^1^ Subsequently, the word ‘papillary’ was dropped in 2005 WHO classification since papillae were not always a prominent finding.^2^ In 2017 WHO classification, the tumor was clubbed as a sub-variant of adenocarcinoma-not otherwise specified (NOS).^3^

Papillary cystadenocarcinoma is one of the uncommon malignancies of the salivary glands accounting for only 2% of all salivary gland lesions.^4^ It is also known as malignant papillary cystadenoma, mucin-producing adenopapillary carcinoma, and low-grade papillary adenocarcinoma.^2^ It most commonly occurs in the parotid gland, followed by the sublingual gland and minor salivary glands.^2^ Papillary cystadenocarcinoma is characterized by cysts and papillary endocystic projections but lacks the features of cystic variants of several common salivary gland carcinomas like polymorphous low-grade adenocarcinoma (PLGA), salivary duct carcinoma, mucoepidermoid carcinoma, and the papillary cystic variant of acinic cell carcinoma.^5^^,^ 6

Herein, we describe a case of papillary cystadenocarcinoma of the parotid gland.

## CASE REPORT

A 54-year-old female presented with the chief complaint of rapid enlargement of the right parotid swelling for eight months. It was associated with pain; however, she did not have any history of fever, cough, or chest pain. General and systemic examinations were normal. On local examination, the swelling was present over the right parotid region measuring 7x5.5 cm. It was firm in consistency. The surface was irregular and have restricted mobility. The overlying skin was unremarkable, with no color change, ulceration, or puckering. No cervical lymph node was palpable.

**Figure 1:**
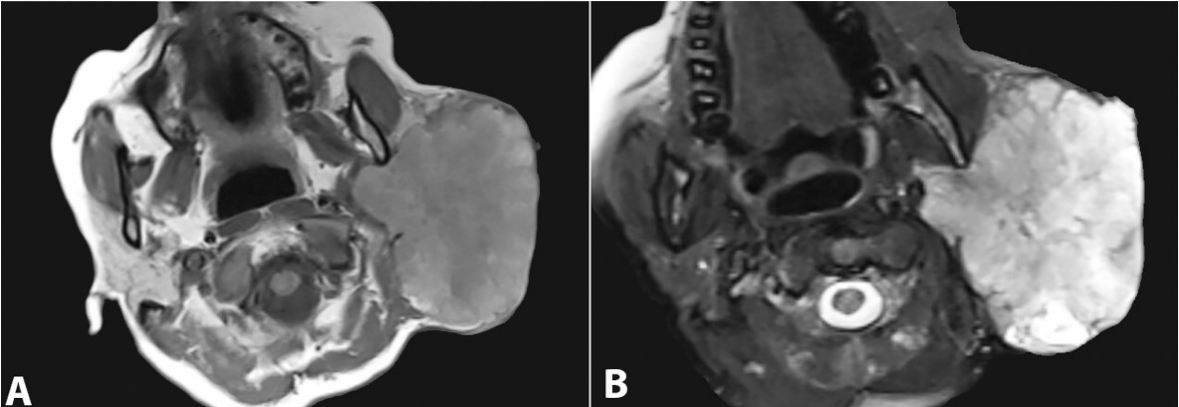
Head and Neck MRI . **A** – T1 weighed image showing a of lobulated parotid mass with hypointense signal; **B** – the mass shows hyperintense signal in T2 weighed image.

Magnetic resonance imaging (MRI) revealed a lobulated mass in the superficial lobe of the right parotid, which was T1 hypointense and T2 hyperintense suggestive of a malignant lesion (Figure 1A and 1B).

Fine-needle aspiration cytology (FNAC) performed from the right parotid swelling was reported as malignant (category VI) as per the “Milan System for Reporting Salivary Gland Cytopathology” (MSRSGC).

**Figure 2:**
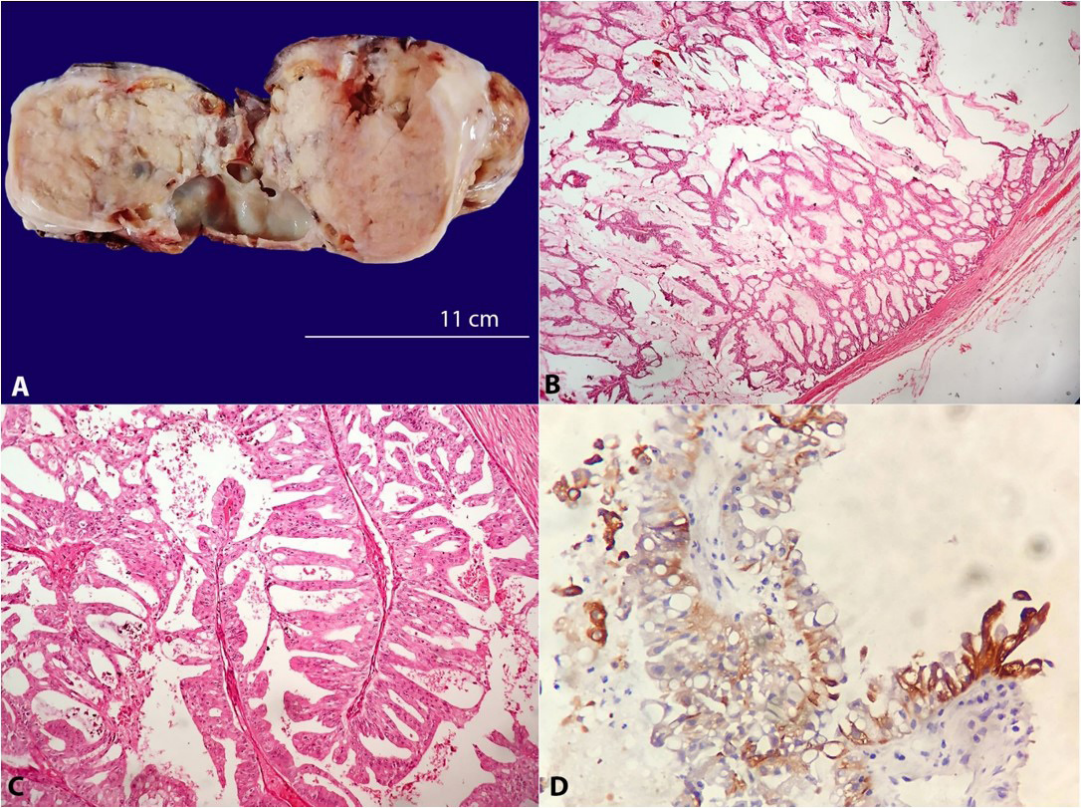
**A** – Gross view of the mass with predominantly solid and scattered cystic areas; **B** – Photomicrograph of the surgical specimen showing complex papillary fronds with fibrovascular cores lined by tall columnar epithelium (H&E, 10X); **C** – The cells exhibited round vesicular eccentric nuclei and a slightly granular cytoplasm. Few cells showed cytoplasmic vacuolation (H&E, 20X); D) CK 7 positivity in the tumor cells (40X).

In view of the malignant nature of the swelling, superficial parotidectomy was done. Grossly, the specimen was circumscribed and globular measuring 8x6x4 cm. At the cut surface, it was mostly solid with a cystic area. The cystic areas were filled with mucoid-like material (Figure 2A). The histopathological examination showed many complex papillary fronds with fibrovascular cores, and lined by tall columnar epithelium. The cells exhibited round vesicular eccentric nuclei and slightly granular cytoplasm. Few cells showed cytoplasmic vacuolation. Intracellular mucin was also noted (Figure 2B and 2C). The tumor cells were positive for CK 7 and CK AE1/AE3 (Figure 2D).

Other markers like CK 20, CK 19, Vimentin, DOG-1, androgen receptor, Her2neu, Gross Cystic Disease Fluid Protein-15 (GCDFP-15), CDX2, and TTF1 were negative. Myoepithelial markers like p63, S-100, Calponin, and Smooth muscle actin (SMA) were negative. The Ki67 index was 8%. In view of these findings, the diagnosis of papillary cystadenocarcinoma of the right parotid was made. The patient was discharged on the 7^th^ post-operative day. There was no locoregional recurrence after 6 months of follow-up.

## DISCUSSION

Salivary gland cystadenocarcinoma is a rare group of epithelial malignancy with indolent biological behavior.^6^ Most salivary gland cystadenocarcinomas occur in the major salivary glands (65%) followed by minor salivary glands (35%).^6^ Among the major salivary glands, the most common site is the parotid glands followed by the sublingual glands.^2^ A search using the keywords ‘papillary cystadenocarcinoma’ and ‘parotid’ in PubMed revealed 36 results. After excluding articles in languages other than English, articles describing papillary cystadenocarcinomas in sites other than parotid glands, and descriptive articles without full or specific information regarding the histologic type or site, 12 articles were included for a review. A similar search in Google Scholar yielded four additional articles, which were included in this review. A total of 16 articles were reviewed comprising 79 cases of papillary cystadenocarcinoma of the parotid gland. (Table 1) 5 ^-^ 20

**Table 1:** Cases of papillary cystadenocarcinoma of parotid gland reported in the English literature

**Ref**	**# cases**	**Age**	**Sex**	**Size (cm)**	**Duration**	**Side**	**Grade**	**IHC**
		**(Y)**						
			M-81.8%	NM	M 10.8 y	NA	NM	Positive CK (KL1 & K8.12), Vimentin & MAM-6;
5	9	a.m 45.7	F-18.2%					Varying intensity for S-100 & NSE
						19 right, 13 left, 3 cases NM	NM	NM
6	35	a.m 58.8	M=F	0.4 to 0.6	1 m - 5 y			
		62	M	3x 2.5	6 m - 14 y	NA	LG	NM
7	2	60	F	4x 2				
8	1	33	F	4x 3	5 m	Left	LG	NM
								Positive CK AE1/AE3 & CEA
9	1	62	M	1.5 X 1.5	NM	NM	LG	Weak positivity for S-100
								Negative Vimentin & SMA
10	1	34	M	6x 6	10 y	Right	LG	NM
11	1	55	M	3	3 m	Right	LG	Positive CK & EMA
								Negative TTF-1
12	1	57	M	6x 5	2 y	Right	LG	Positive CK7, EMA & S-100
								Negative CEA, SMA, GFAP, ER, PR & Her2neu
13	1	40	F	1.5X0.5	10 y	Right	LG	NM
14	1	33	F	4x 3	5 m	Left	LG	NM
15	1	58	M	5x4x2	4 m	Right	NM	NM
16	1	55	M	6 × 5	3 y	Right	NM	Positive CK7
								Negative CK20, p63 & Cerb2
17	1	47	M	9x6. 5x3	4 y	Right	IG	NM
18	1	51	M	3.3 × 3.2 × 2.8	4 y	Right	HG	NM
		a.m 49.55	M-66.7%	<3-29.6%	≤ 12 m- 59.3%	Right-55.6%	NM	NM
			F-33.3%	≥3- 70.4%	13-36 m-25.9%	Left-44.4%		
19	19				>36 m- 14.8%			
20	1	67	F	6x 5 cm	6 months	Right	LG	NM	

a.m= arithmetic mean; F= female; HG= high grade; IG= intermediate grade; LG= low grade; m= month; M= male; NM= not mentioned; y= year.

Papillary cystadenocarcinoma is usually a slow-growing, compressible asymptomatic mass.^3^ Around 71% of the patients are over 50 years of age.^6^ Although no sex predilection has been documented by Foss et al.^6^ for salivary gland PCAC, an analysis of the parotid papillary cystadenocarcinoma shows most cases are documented in males with almost all cases presenting over 50 years of age. (Table 1) The present case was a female patient and was over 50 years of age at the time of presentation.

MRI is critical in determining the nature of the lesion, whether solid, cystic, or necrotic, as well as its relationship to the salivary gland, adjacent structures, and the extent of infiltration due to its excellent spatial resolution and superior soft-tissue contrast.^21^ The present case had both solid and cystic areas and infiltrated the adjacent structures.

The diagnostic accuracy of pre-operative FNAC of salivary gland tumors ranges from 80-95%.^8^ However, the exact typing of salivary gland tumors on FNAC often poses a problem due to cytological overlap.^8^The present case was typed as malignant (category VI) as per the MSRSGC.

Microscopically papillary cystadenocarcinoma of the salivary gland is characterized by cystic and solid areas with multiple luminal papillary projections having a fibrovascular core and lined by cuboidal, columnar, or mucus-secreting epithelial cells.^2^^,^ 4 ^,^ 6 The tumor cells show mild to moderate nuclear atypia with one or two small distinct hyperchromatic nucleoli and eosinophilic to vacuolated cytoplasm.^2^^,^ 4 ^,^ 6 Papillary cystadenocarcinoma must be distinguished from its benign counterpart cystadenoma based on the former’s infiltrative growth pattern into adjacent surrounding tissues and atypical nuclear features.^6^

The closest differential diagnoses include PLGA, papillary cystic variant of acinic cell carcinoma, salivary duct carcinoma, and mucoepidermoid carcinoma.^13^^,^ 22 The various morphologic and immunohistochemical characteristics of these entities are summarized in Table 2.

**Table 2:** Differentiating features (histomorphology and immunohistochemistry) of the differential diagnoses of papillary cystadenocarcinoma

**Entity**	**Most common site**	**Tumor architecture**	**Tumor cells**	**Cytokeratin**	**Calponin/** **SMA**	**Other IHC markers**
PLGA	Minor salivary glands	Variable patterns of different proportions including trabecular, tubular, papillary, solid and cribriform patterns	tumor cells having pale nuclei with marked chromatin clearing	Positive for CK7, CK AE1/AE3	Variable	Positive for Vimentin and S-100
Papillary cystic variant of acinic cell carcinoma	Parotid glands	Transition of usual dense cellularity into papillary folds interspersed with cystic spaces.	Diverse tumor cell types including acinic cells, vacuolated cells, intercalated cells, non-specific glandular cells forming ‘hobnail cells’ and clear cells	Positive for CK7	Negative	Positive for DOG-1
Salivary duct carcinoma	Parotid glands	Invasive tumor forming cords, nests and cribriform glands in a desmoplastic stroma	Tumor cells have ample eosinophilic cytoplasm showing marked nuclear pleomorphism	Positive for CK7, CK AE1/AE3	Negative	Positive for Androgen receptor, Her2neu, and GCDFP-15
Muco epidermoid carcinoma	Parotid glands	Incompletely encapsulated or unencapsulated tumor showing papillary or glandular growth pattern	Varying proportions of Squamoid, intermediate cells, and mucophages	Positive for CK7, CK19	Negative	Positive for p63
Papillary cystadenocarcinoma	Parotid glands	Cysts with papillary endocystic projections	Cuboidal to tall columnar cells with vesicular nuclei and slightly granular cytoplasm	Positive for CK7, CK AE1/AE3	Negative	Negative for S-100, DOG-1, AR, Her2neu, GCDFP-15, p63

The other uncommon differential diagnoses are mucinous adenocarcinoma and intestinal-type adenocarcinoma, and metastatic papillary carcinoma of the thyroid. 2 ^,^ 3 Papillary carcinoma of the thyroid shows characteristic nuclear features and expresses nuclear positivity for thyroid transcription factor 1 (TTF-1). Mucinous adenocarcinoma and intestinal-type adenocarcinoma are two other uncommon subtypes of adenocarcinoma-NOS, which need to be differentiated from papillary cystadenocarcinoma as these tumors have an aggressive clinical course.^3^ Histopathologically, mucinous adenocarcinoma is characterized by large pools of extracellular mucin and immunopositive for CK 7. Intestinal-type adenocarcinoma is positive for CK 20 and CDX2.^2^^,^ 3

In the present case, characteristic morphology showing multiple luminal papillary projections having fibrovascular cores, which were lined by tall columnar and mucinous cells, pointed to a diagnosis of papillary cystadenocarcinoma. Immunopositivity for CK 7 and CK AE1/AE3, and negativity for other epithelial, myoepithelial, and specific markers for other salivary tumors were consistent with papillary cystadenocarcinoma.^12^^,^ 23

Salivary gland papillary cystadenocarcinoma is a low-grade tumor with an indolent clinical course.^4^ However, there are isolated cases of salivary papillary cystadenocarcinoma with aggressive behavior in the form of high mitotic activity and cervical nodal metastasis.^24^ The treatment of choice is complete surgical excision (superficial parotidectomy).^12^ High-grade tumors require neck dissection. 24

The present case describes a rare sub-variant of adenocarcinoma-NOS of the salivary gland, which is no more included in the newer WHO classification. However, it is important to report all rare sub-variants in order to understand their biology and behavior.
